# An impaired metabolic response to hydrostatic pressure explains *Alcanivorax borkumensis* recorded distribution in the deep marine water column

**DOI:** 10.1038/srep31316

**Published:** 2016-08-12

**Authors:** Alberto Scoma, Marta Barbato, Sara Borin, Daniele Daffonchio, Nico Boon

**Affiliations:** 1 Center of Microbial Ecology and Technology (CMET), University of Gent, Coupure Links 653, B 9000 Gent, Belgium; 2 Department of Food, Environmental and Nutritional Sciences (DeFENS), University of Milano, Via Celoria 2, 20133, Milano, Italy; 3 Biological and Environmental Sciences and Engineering Division, King Abdullah University of Science and Technology (KAUST), 4700, Thuwal 23955-6900, Kingdom of Saudi Arabia

## Abstract

*Alcanivorax borkumensis* is an ubiquitous model organism for hydrocarbonoclastic bacteria, which dominates polluted surface waters. Its negligible presence in oil-contaminated deep waters (as observed during the Deepwater Horizon accident) raises the hypothesis that it may lack adaptive mechanisms to hydrostatic pressure (HP). The type strain SK2 was tested under 0.1, 5 and 10 MPa (corresponding to surface water, 500 and 1000 m depth, respectively). While 5 MPa essentially inactivated SK2, further increase to 10 MPa triggered some resistance mechanism, as indicated by higher total and intact cell numbers. Under 10 MPa, SK2 upregulated the synthetic pathway of the osmolyte ectoine, whose concentration increased from 0.45 to 4.71 fmoles cell^−1^. Central biosynthetic pathways such as cell replication, glyoxylate and Krebs cycles, amino acids metabolism and fatty acids biosynthesis, but not *β*-oxidation, were upregulated or unaffected at 10 MPa, although total cell number was remarkably lower with respect to 0.1 MPa. Concomitantly, expression of more than 50% of SK2 genes was downregulated, including genes related to ATP generation, respiration and protein translation. Thus, *A. borkumensis* lacks proper adaptation to HP but activates resistance mechanisms. These consist in poorly efficient biosynthetic rather than energy-yielding degradation-related pathways, and suggest that HP does represent a major driver for its distribution at deep-sea.

Enhanced microbial hydrocarbons degradation represents one of the most important remediation strategies for marine petroleum contamination. While the use of booms with skimmers and dispersants may account for recovering or dissolving the largest fraction of the spilled oil^1^, the last, fine hydrocarbons removal step relies on bacterial degradation. Soon after oil is spilled, microbial community structures on surface waters are largely modified^2^ and members of the genus *Alcanivorax* frequently dominate such bacterial blooms accounting for more than 80% of the total bacterial population^3^,^4^,^5^. Such a prominent role in petroleum-affected environments is due to some critical features possessed by *Alcanivorax*, including the ability to efficiently use branched-chain alkanes^6^ and the capacity to enhance the bioavailability of hydrophobic compounds in water. Further, in hydrocarbonoclastic bacteria utilization of carbon sources alternative to oil is limited to few metabolic intermediates, such as acetate and pyruvate, making them very selective towards hydrocarbons^6^,^7^. The *Alcanivorax* genus was initially described by Yakimov and co-workers in 1998, who isolated *A. borkumensis* SK2 and proposed it as the type strain. It was later recognized that this ubiquitous genus, and *A. borkumensis* in particular, dominates oil-contaminated surface marine waters all over the world^8^,^9^, background to why *A. borkumensis* SK2 is being today adopted as a model organism to investigate hydrocarbon degradation pathways in marine environments^10^.

A limitation to oil bioremediation is the tendency of oil to create tar balls and droplets, which eventually sink to the seafloor together with bacterial biomass belonging to the surface^11^. Overwhelming oil release to the environment enhances also marine snow formation^12^, which has been recognized as the main driver for seafloor contamination in the Deepwater Horizon (DWH) spill in April 2010^13^. These phenomena postulate that microbial oil degraders at the sea surface will eventually deal with increased hydrostatic pressure (HP) typical of the deep sea. Oil in the deep sea also results from the release by natural seeps or following the use of dispersants^14^, by adsorption to heavier particulate or non-miscible components, or due to problems encountered at deep-sea drilling sites as in the case of the DWH spill^15^. Studies on the fate of the deep oil plume occurring after the DWH spill indicated that bacteria other than *Alcanivorax* were mainly enriched during hydrocarbon biodegradation^16^,^17^,^18^,^19^,^20^,^21^. Several environmental factors such as low temperature or lack of nutrients were proposed as conditional for the enrichment of *Alkanivorax* in the deep water oil plume^22^, but supporting evidence was not supplied^20^. On the contrary, to our knowledge the HP occurring at the depth of the DWH spill has been neglected as a possible factor to explain the low abundance of *Alcanivorax* detected after the oil spill. Considering the ubiquity of *Alcanivorax* in polluted surface marine waters, including those in the Gulf of Mexico after the DWH oil spill^23^, the low frequency of *Alcanivorax* in the DWH oil plume in the deep water column raises the hypothesis that this organism may not effectively respond to HP. Our hypothesis is that mild HP (up to 10 MPa, equivalent to1 km in the marine water column, approximately the depth of the DWH oil spill) may be sufficiently stressing to exert an impact on *A. borkumensis* metabolism and potentially impair its remarkable oil-bioremediation capacity. In the present study, *A. borkumensis* SK2 physiological response to 0.1, 5 and 10 MPa grown on the alkane *n*-dodecane was compared, and integrated with information derived from the analysis of the transcriptome at 0.1 and 10 MPa.

## Results

### Growth yields under atmospheric and increased HP in A. borkumensis SK2

**Figure 1:**
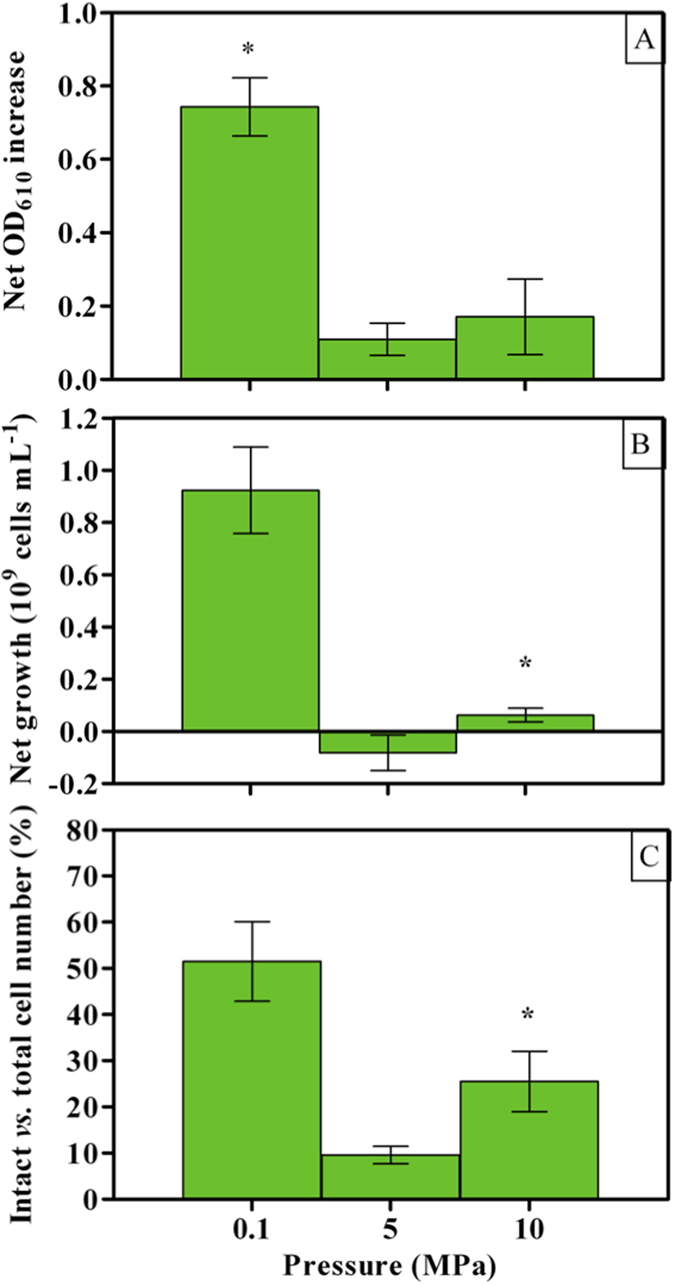
Culture growth of *A. borkumensis* SK2 cells under different HPs (0.1, 5 and 10 MPa). (**A**) Net optical density increase; (**B**) Net cell number increase; (**C**) final cell integrity. Bars indicate 95% confidence intervals. Asterisks indicate statistical significance (*P* < 0.05), that is: in (**A**), mean average value at 0.1 MPa is higher than at 5 and 10 MPa; in (**B**), mean average values at 10 MPa are higher than at 5 MPa.

Cell replication and metabolism in *A. borkumensis* cultures were examined under atmospheric (0.1 MPa) and mild (5 or 10 MPa) HP. Change in OD_610_ was substantial under atmospheric pressure, but dramatically decreased under 5 and 10 MPa (*P* < 0.05; Fig. 1A) as also observed for total (Fig. 1B) and intact cell number (Fig. 1C). However, both these latter values were significantly higher under 10 MPa than 5 MPa (*P* < 0.05; Fig. 1B,C) indicating that some pressure-resistance mechanism was activated over the stressing HP of 5 MPa. Dodecane concentration was evaluated by analyzing its solubilized fraction in the culture broth at the end of the incubation (Fig. S1). This analysis aimed at understanding whether experimental conditions limited the access to the supplied carbon source, provided that dodecane solubility in saline waters is lower than 2 μg L^−1^ 24. Notwithstanding the very different cell number (Fig. 1B), comparable values were found in cultures incubated under 0.1 and 10 MPa (Fig. S1), suggesting that access to dodecane was not a limiting factor. The highest values of solubilized dodecane were observed under 5 MPa (Fig. S1) despite cells did not grow (Fig. 1B). This may be explained with an increasingly impaired metabolism in SK2 during incubation under HP, where solubilized dodecane is eventually not taken up. Full understanding of this mechanism requires further experimental evidence, the outcome of which would be out of the scope of the present investigation.

**Figure 2:**
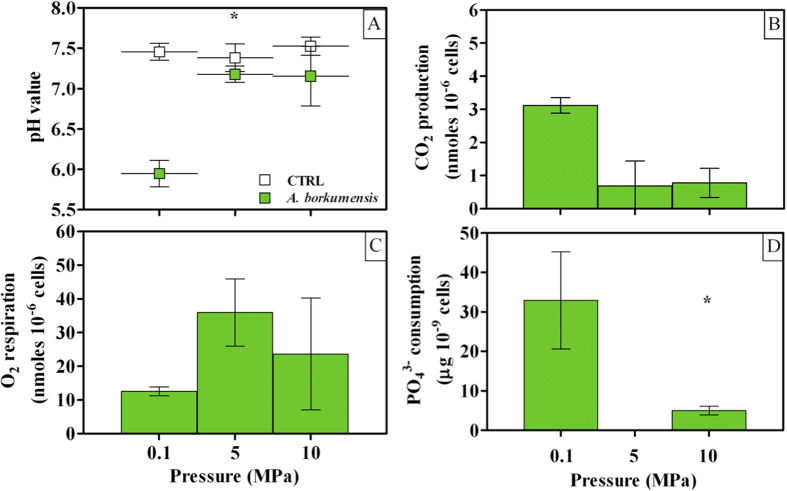
Cell metabolism in *A. borkumensis* SK2 cells grown under different HPs (0.1, 5 and 10 MPa). (**A**) pH decrease with respect to sterile controls (keys reported in the graph); (**B**) CO_2_ production per cell; (**C**) O_2_ respiration per cell; (**D**) uptake of PO_4_^3−^. Bars indicate 95% confidence intervals. Asterisks indicate statistical significance (*P* < 0.05), that is: in (**A**), mean average value at 0.1 MPa is lower than at 5 and 10 MPa; (**B**), mean average value at 0.1 MPa is higher than at 10 MPa.

Since dodecane was the sole carbon source supplied to *A. borkumensis* cells, its degradation could be estimated by following pH decrease and measuring CO_2_ production with respect to sterile controls. Sustained medium acidification was detected under 0.1 MPa (Fig. 2A) in agreement with a higher OD_610_ and cell number (Fig. 1A,B, respectively), while little difference in pH was observed under 5 and 10 MPa as compared to sterile controls (Fig. 2A). Variations in pH were mirrored by CO_2_ production, as the latter significantly decreased under mild HP to low, comparable values at 5 and 10 MPa (*P* > 0.05, Fig. 2B). Conversely, respiration capacity was generally enhanced by increased HP (Fig. 2C). The lack of a linear relation between CO_2_ production (Fig. 2B) and O_2_ respiration (Fig. 2C) when comparing atmospheric and mild HP may be considered as an indication of the shift in cell metabolism under increased HP. PO_4_^3−^ uptake was completely inhibited when increasing HP to 5 MPa, although some activity was restored with further HP increase to 10 MPa (Fig. 2D).

As a whole, a mild HP increase to 5 MPa was lethal for *A. borkumensis* SK2 while doubling such a HP re-established some cell replication and improved cell integrity and activity, indicating that some pressure-resistance mechanism was triggered. To ascertain this hypothesis the transcriptomic response of cells grown under 0.1 and 10 MPa was compared.

### Transcriptomic response in A. borkumensis SK2 cells under 0.1 and 10 MPa

Application of 10 MPa HP resulted into a reduced expression of the majority of the genes (56%, 1242/2202), while only a minor fraction was upregulated (16%, 354/2202) and the rest remained unaffected (28%, 606/2202). Increased expression involved various clusters of orthologous genes (COG), the most represented of which were protein translation, ribosomal structure and biogenesis, amino acid and coenzyme metabolism, energy production and transcription (Fig. S2). Several genes that are not presently categorized in any COG were also upregulated (Fig. S2).

### Fatty acids and alkane metabolism

Genes related to fatty acids metabolism were generally upregulated or showed the same expression level at 10 MPa as at ambient pressure (6/11, Table 1), and in particular enzymes related to alkanes activation (such as cytochrome P450 and the alkane 1 mono-oxygenase, Table 1). However, biosynthesis of fatty acids rather than *β*-oxidation appeared to be triggered by HP. In fact, genes related with fatty acids degradation were generally downregulated (6/9, Table 1) while those expressing enzymes linked with fatty acids biosynthesis were more highly expressed or remained unaffected (6/7, Table 1). An alternative pathway to produce energy with fatty acids is the glyoxylate cycle, whose genes were either more highly expressed under HP or remained unaffected (6/7, Table 2). The glyoxylate cycle generates oxalacetate and is strictly connected to several pathways among which the Krebs (or tricarboxylic acid [TCA]) cycle^25^. In the latter, the large majority of the genes was either upregulated or remained unaffected under HP (18/21, Table 2). Other pathways involved in the production of energy, such as glycolysis-gluconeogenesis and the pentose phosphate pathway, were generally downregulated (data not shown). However, gene expression of the enzymes connecting the TCA cycle to the purine, pyrimidine and histidine metabolism through glycolysis/gluconeogenesis and the pentose phosphate pathway were either upregulated or unaffected (Table S1), suggesting that under HP these pathways may have been served with metabolic intermediates to support the production of nucleotides.

### Amino acids and derivate compounds

**Figure 3:**
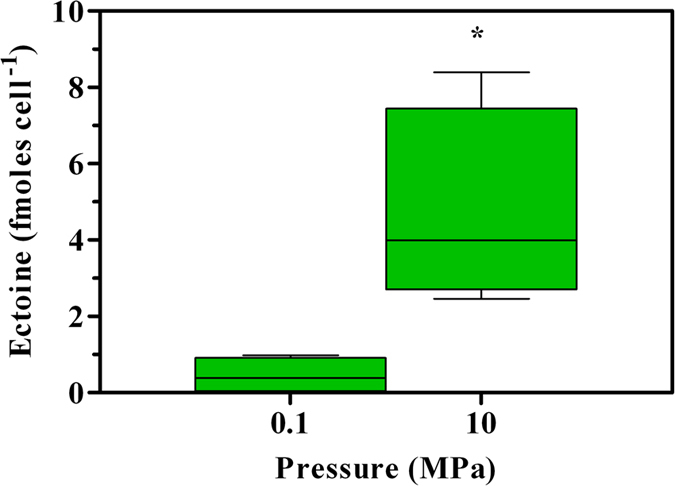
Accumulation of intracellular ectoine per cell in *A. borkumensis* SK2 grown under different HPs (0.1 and 10 MPa). Bars indicate 95% confidence intervals. Asterisk indicates that values obtained under 10 MPa were significantly higher (*P* < 0.05) than those observed under 0.1 MPa.

Several pathways related to the synthesis of amino acids were upregulated. Genes involved in the metabolism of glycine, serine and threonine (15/16, Table 3), biosynthesis of L-leucine (10/10, Table 3), and biosynthesis of valine, leucine and isoleucine (11/13, Table 3) were either upregulated or remained unaffected, together with the majority of those supporting cell division (6/11, Table 3). As a notable exception, the whole pathway leading to the production of the nitrogen-based osmolyte ectoine was upregulated (Table 4). Analysis of the different biomasses grown at 0.1 and 10 MPa confirmed a remarkable 10-fold increase in the amount of ectoine produced per cell in cultures grown under HP (from 0.45 to 4.71 fmoles cell^−1^, respectively; *P* < 0.05; Fig. 3).

### Respiration and ATP generation

Expression of almost all the genes involved in the formation of the ATP synthase complex was upregulated (8/9, Table 2), indicating that HP may have deeply affected such multimeric enzyme and energy production. Genes involved in respiration were also highly impacted by HP. The large majority of the genes encoding for cytochrome c oxidases (6/9, Table 2) and cytochrome b (1/1, Table 2) were downregulated, the latter being the most downregulated gene involved in respiration. On the contrary, cytochrome c reductases were either upregulated or remained unaffected (3/3, Table 2). Furthermore, the majority of the genes expressing subunits of the Na^+^-translocating NADH-quinone reductase were either upregulated or unaffected (5/6, Table 2), supporting the hypothesis that the microbial respiration chain under HP may follow different pathways with respect to that occurring at ambient pressure.

### Transcription and translation

The transcriptional machinery appeared to be only slightly impacted by HP. While genes coding for transcriptional regulators generally showed a lower expression level (29/46, Table S2), 5/8 genes coding for elongators, activators and termination factors were upregulated (Table S2). Similarly, genes involved in DNA replication and repair were only partially expressed at a higher level under HP (1/10 and 2/9, respectively, Table S3). On the contrary, protein translation was highly affected, as several translation (4/6) and elongation factors (3/3) were upregulated (Table S4). All RNA polymerase genes (4/4, Table S4) were upregulated, while the tRNA regulator pseudouridine synthase was either upregulated or unaffected (3/3, Table S4). Notably, genes encoding for ribosomal proteins were largely upregulated (45/53, Table S5), suggesting that mild HP deeply affected this multicomponent protein complex. On the contrary, some of the typical HP-responsive pool of genes (such as sigma factors, chaperonins and outer membrane proteins) were marginally affected by HP increase (Table S6).

## Discussion

Worldwide, microbial communities developing on oil-contaminated surface waters were dominated by *A. borkumensis,* which grows rapidly right after oil spills^10^,^26^,^27^. In deep-sea waters *A. borkumensis,* and in general the genus *Alcanivorax,* is much less common according to studies following the actual enriched microbial community developed after the DWH oil spill at depths of 1000–1300 m^16^,^17^,^18^,^19^,^20^. *Alcanivorax* signatures in the DWH case studies were reported in sediments at a low relative abundance (only few tens of the *Oceanospirillales* sequences that represented just 1.4–1.7% of total bacterial sequences) and it was concluded that they did not correlate with the hydrocarbon levels associated with the sample^28^. Gutierrez and co-workers identified *A. borkumensis* SK2-like sequences in deep waters from the DWH oil spill^20^. However, according to sequence abundance they considered *Alcanivorax* contribution to oil degradation rather negligible^20^. They also obtained *Alcanivorax* isolates with enrichment experiments performed on decompressed samples, thereby eliminating a specific environmental factor of the DWH plume^20^. This methodology has been typically applied to the known *Alcanivorax* species isolated from deep-sea environments^29^,^30^,^31^. Our findings indicate that *A. borkum*ensis SK2 might not be enriched under the mild HP of the DWH deep waters, suggesting that the lack of such a selection factor would restore the capacity of *Alcanivorax* to grow. The role of HP on *A. borkumensis* physiological and molecular response has never been investigated, leaving a knowledge gap about its role, deep-sea distribution and activity. Our experiments on strain SK2 indicate that it is a piezosensitive microbe and that mild HP remarkably affects *A. borkumensis* growth and physiology, explaining its low abundance in the sea water column. While temperature may also play a role in determining such a low abundance in deep-sea environments, it must be noted that strain SK2 does grow with temperatures as low as 4 °C^7^.

The stoichiometric mineralization of dodecane would yield a maximum CO_2_:O_2_ molar ratio of 0.649, as 12 moles of CO_2_ would be generated from 1 mole of dodecane using 18.5 moles of O_2_ (Eq. 1).


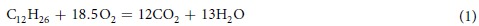


As part of the supplied dodecane may be used to build up microbial biomass through some metabolic intermediates, Eq. 1 overestimates the effective mineralization rate and it rather represents the upper limit for dodecane degradation by the cells. Growth yields were high under surface-water-resembling conditions (0.1 MPa; Fig. 1A,B). Coherently, dodecane biodegradation rates were about 1/3 of the stoichiometric conversion ratio (0.248 *vs.* 0.649, Eq. 1). Application of a HP equivalent to 5 MPa was lethal, as it essentially inactivated SK2: final cell number was lower than what initially inoculated, decreasing to almost undetectable values (58 ± 47*10^6^ cells mL^−1^) with more than 90% of such surviving cells being damaged (Fig. 1C). Uptake of PO_4_^3−^ was completely inhibited (Fig. 2D), while O_2_ respiration increased (Fig. 2C) and CO_2_ production dropped (Fig. 2B), resulting in a CO_2_:O_2_ molar ratio of 0.019. The lack of a significant difference (*P* > 0.05) in pH value between sterile controls and cells incubated under 5 MPa (Fig. 2A) is a good indication of the effects of HP on the metabolic potential of *A. borkumensis* SK2. However, application of HPs twice as high as this lethal one triggered some HP-resistance mechanism. While the bioremediation potential remained as low as that observed at 5 MPa (Fig. 2A), under 10 MPa some culture growth was re-established (Fig. 1B), cell integrity improved (Fig. 1C), CO_2_:O_2_ molar ratio slightly increased (0.033) and uptake of some critical nutrients restored (Fig. 2D).

### HP resistance conferred by the piezolyte ectoine

Enhanced bacterial fitness upon further HP increase to 10 MPa was consistent with the synthesis of the osmolyte ectoine (Table 4 and Fig. 3). Several organic and inorganic solutes accumulate intracellularly under osmotic and thermal stress to maintain turgor pressure, cell hydration or stabilize macromolecular structures, while little is known about solutes implicated in counteracting the destabilizing effects exerted by HP^32^. Ectoine is a very well known nitrogen-based organic osmolyte^33^ produced in response to an increased salinity^33^,^34^, and has been found in genera that include several piezophilic species such as *Vibrio* and *Photobacterium*^35^. However, previous studies failed to observe accumulation of ectoine under increased HP in both piezophilic^36^ and piezosensitive bacteria^37^. Hence, the present study is the first describing ectoine as a piezolyte, a class that includes solutes accumulated under both osmotic and HP. The exact protective mechanisms exerted by ectoine are not clear as well as the triggering effect leading to its enhanced production. In principle, HP does not result into a pressure difference across the membrane rather in destabilization of macromolecules^32^. Observation that other piezosensitive bacteria such as *A. dieselolei* KS 293 do not upregulate synthetic pathways under 10 MPa^38^ leads to two hypothesis: 1) either ectoine synthesis under HP is a peculiar response of *A. borkumensis* cells or, more likely, 2) critical thresholds for membrane integrity exist, as in *A. dieselolei* KS 293 intact cell number was almost unaffected at 0.1 and 10 MPa (about 70%^38^). However, non-linear responses between HP and cell integrity have already been reported^39^, therefore the possibility that other mechanisms may (co-)regulate ectoine production should be thoroughly investigated.

Provided that the averaged bacterial cell dry weight (CDW) is 10^−12^ g^40^,^41^, estimates of the highest ectoine accumulation capacity in *A. borkumensis* SK2 cells at 10 MPa would yield 0.59 g_ectoine_ g^−1^_CDW_. This value is higher than what reported with some of the most productive strains in the literature (*Halomonas salina*^42^*, Brevibacter linens*^43^ and *B. epidermis*^44^ yielding 0.35, 0.21 and 0.16 g_ectoine_ g^−1^_CDW_, respectively), cultivated at ambient pressure under increased salinity (between 0.5 and 1 M NaCl^42^,^43^,^44^ *vs*. 0.4 M of the present study). However, owe to SK2 limited growth yields at 10 MPa (Fig. 1B), ectoine concentration in the culture broth was rather low (0.12 g_ectoine_ L^−1^), while much higher productivities could be achieved in *H. salina*^42^ and *B. epidermis*^44^ (6.9 and 8 g_ectoine_ L^−1^). The apparently counter-productive investment in the energy-intensive^43^ *de novo* ectoine synthesis under the stressing conditions of 10 MPa (Figs 1C and 2D) may be considered as a good indication of SK2 limited capability to adapt to HP.

### Alkane and fatty acids metabolism

Genome expression was largely suppressed by exposure to 10 MPa, confirming a general deleterious effect on SK2 metabolism and its piezosensitive nature. However, unaffected and upregulated pathways described an integrated response aimed at supporting cell replication. Genes involved in alkanes activation^45^ were upregulated under HP (Table 1). Introduction of an oxygen atom into saturated hydrocarbons may proceed through a terminal or subterminal pathway, which can coexist within the same microorganism^46^. In the present study this would result into the generation of dodecanoyl-CoA (terminal oxidation) and/or decanoyl-CoA and acetyl-CoA (subterminal oxidation). While the exact mechanisms of alkane activation and the generated metabolic intermediate remain unknown, it appears that once such fatty acids were introduced into cell metabolism their elongation rather than their degradation was preferred under 10 MPa (Table 1). None of the genes associated with *β*-oxidation was upregulated (0/9, Table 1), contrary to what observed with fatty acids biosynthesis (4/7, Table 1). Reduced expression of genes related with *β*-oxidation under HP does not exclude that this pathway may have been active to some extent. However, *A. borkumensis* SK2 is also known to be able to withdrawn oxidized *n*-alkanes from the degradation pathway and incorporate them as their corresponding fatty acids in the membrane^47^, a mechanism already observed in other hydrocarbon-degrading microbes (*e.g., Marinobacter hydrocarbonoclasticus*^48^ and *Rhodococcus erythropolis*^49^). The main fatty acid components in *A. borkumensis* membrane range between C14 and C18 7, therefore their elongation would be needed prior to incorporation. Strain SK2 does possess the capacity to elongate *n*-alkane-oxidized fatty acids by addition of C2 units^47^. Membranes represent 10% of bacterial biomass^47^, and using this pathway would avoid synthesizing fatty acids *de novo*, thus representing an effective energy-saving strategy under stress conditions.

Nonetheless, alternative pathways related with energy generation also making use of fatty acids were upregulated, such as the glyoxylate and TCA cycle. These pathways share several genes and metabolic intermediates and were both found to be active under HP (Table 2). The glyoxylate cycle is the main biosynthetic route starting from fatty acids^25^ serving several other pathways such as glycine synthesis starting from glyoxylate itself 50. Enzymes related with glycine synthesis are interconnected with serine and threonine metabolism^51^, the synthesis of all of the three being enhanced under HP (Table 3). As such, upregulated genes involved in cell division (Table 3) would be potentially served with energy (glyoxylate and TCA cycles [Table 2]) and some of the major building blocks to produce bacterial biomass (fatty acids through reverse *β*-oxidation [Table 1] and amino acids [Table 3]). Further, the biotin synthesis pathway was found to be active under HP (Table S7). Generation of this cofactor takes advantage of metabolic intermediates derived from fatty acids biosynthesis^52^, which was enhanced under HP (Table 1).

Notwithstanding the enhanced expression under mild HP of genes related to biosynthetic pathways, the final number of cells at 10 MPa was markedly lower than that measured at 0.1 MPa (Fig. 1B). This may indicate that the actual activity of the related enzymes may have been compromised under HP, or that other key pathways supporting cell replication were critically impaired. This could be the case of ATP generation and protein translation.

### ATP generation and alternative respiration pathways

Mild HP may have deeply affected ATP generation in strain SK2, as almost all ATP synthase subunits were upregulated under 10 MPa (Table 2). Energetic hurdles due to extreme, stressing conditions are known to impact ATP intracellular balance at high pH^53^ and salinity^54^, with acid^55^ or HP stress (about 40 MPa^56^). Although increased cell damaging (Fig. 1C) may have contributed significantly to raise the energy requirement for cell maintenance, PO_4_^3−^ uptake per cell did not increase between 0.1 and 10 MPa (Fig. 2). In this perspective, enhanced biosynthesis rather than degradation of fatty acids (Table 1) may be part of an energy-saving strategy, where incorporating *n*-alkane-oxidized fatty acids^47^ to build up cell components would be more convenient than degrading dodecanoyl- or decanoyl-CoA to di- or tri-carboxylic acids and synthesize them again through the glyoxylate and TCA cycle, especially when ATP generation is impaired. As concerns respiration, mild HP shifted cytochrome c species from oxidases to reductases (Table 2) and downregulated the expression of cytochrome b. Accordingly, genes related with several Na^+^-translocating quinone reductase subunits were more highly expressed (Table 2). A similar response to mild HP in oil-degrading *Alcanivorax* species was previously observed^35^. In agreement, the present data support the hypothesis that mild HP (10 MPa) could induce alternative respiration pathways as those proposed in *Shewanella benthica* under high HP (60 MPa^57^,^58^). As it stands, the increased HP impact on *A. borkumensis* cells (Fig. 1B,C) as compared to *A. dieselolei*^35^ resulted into a larger and higher level of expression of genes belonging to these alternative respiration pathways and ATP generation (Table 2).

### DNA transcription, synthesis and repair, and protein translation

DNA integrity and synthesis was not compromised by mild HP (Table S3), while several transcriptional regulators, elongators and termination factors were upregulated (Table S2), likely serving cell replication purposes. Concerning protein translation, this is one of the most HP-sensitive processes. Aminoacyl-tRNA binding to ribosomes determines a conformational change in the latter that leads to an increase in volume^59^. As processes determining a volume increase are not favored under HP^60^, protein synthesis efficiency and accuracy is slowed down or impaired by high HP (as tested between 55 to 400 MPa^61^,^62^,^63^,^64^,^65^). Expression of almost all ribosome subunits was upregulated under 10 MPa (Table S5) together with translation, elongation and tRNA modifying factors such as the pseudouridine synthase (Table S4). These results are consistent with the response observed with other hydrocarbonoclastic piezosensitive bacteria subjected to 10 MPa^35^, indicating that protein synthesis is highly impacted already under mild HP. Maintenance of functional multicomponent proteins and organelles such as ribosomes^32^ may play a major role in the development of microbial community structures with enhanced bioremediation potential.

## Materials and Methods

### Strain, culture media and growth conditions

*Alcanivorax borkumensis* SK2 was kindly provided by Prof. Fernando Rojo (CSIC, Spain), and cultivated axenically in static glass bottles of 250 mL (operating volume 100 mL), using ONR7a medium^66^, initial pH 7.6, for 4 to 7 days at 20 °C. Cultures were provided with 1% (v:v) *n*-dodecane (Sigma Aldrich, Belgium) as sole carbon source (equivalent to about 7.5 g L^−1^) in order to imitate the conditions of an oil spill (high C/N ratio) as previously suggested with this strain^39^.

### Mild HP experiments

Early stationary phase cells were collected by centrifugation at 4000 rpm for 10 min at 4 °C (Sorval RC5c PLUS, Beckman, Suarlée, Belgium) and resuspended in fresh ONR7a medium at an initial optical density (OD_610_) of 0.100 ± 0.005, corresponding to 140 ± 30 × 10^6^ cells mL^−1^. Then, 3.5 mL of culture suspension was transferred into sterile 10 mL syringes and *n*-dodecane (C12) 1% (v:v) supplied as the sole carbon source. Gas phase (equal to 6.5 mL) was constituted of air, which provided O_2_ to the cells during the subsequent incubation. Syringes were closed using a sterile three-way valve, and placed in a 1L T316 stainless steel high-pressure reactor (HPR) (Parr, USA). The reactor was filled with deionized water and HP was increased up to 5 or 10 MPa by pumping water with a high-pressure pump (HPLC pump series III, SSI, USA). Pressure was transmitted to the cultures through the piston of the syringe. Experiments at atmospheric pressure were run adjacent to the HPR. Control experiments were constituted by sterile syringes supplied only with sterile non-inoculated medium. Reactors were incubated in a temperature-controlled room at 20 °C for 4 days reaching the stationary phase. At the end of the experiments, pressure was gently released and syringes set aside for 30 min before running biochemical analyses, unless otherwise specified.

### Cell counts and related analyses

Optical density was measured at 610 nm (OD_610_) with a spectrophotometer (Isis 9000, Dr Lange, Germany). Pressure-induced cell membrane damaged analysis and total cell count was adapted after^67^ using flow cytometry: SYBR^®^ Green I and Propidium Iodide were used in combination to discriminate cells with intact and damaged cytoplasmic membranes using a protocol previously described^68^.

### Chemical analyses

O_2_ respiration and CO_2_ production rates were assessed by comparing the headspace biogas composition of syringes inoculated with strain SK2 cells and sterile controls. Gas-phase was analyzed with a Compact GC (Global Analyser Solutions, Breda, The Netherlands), equipped with a Molsieve 5A pre-column and two channels. In channel 1, a Porabond column detected CH_4_, O_2_, H_2_ and N_2_. In channel 2, a Rt-Q-bond pre-column and column detected CO_2_, N_2_O and H_2_S. Biogas concentrations were determined with a thermal conductivity detector. pH was measured using a pH meter (Herisau, Metrohm, Switzerland). Phosphate was quantified with a Compact Ion Chromatograph (Herisau, Metrohm, Switzerland) equipped with a conductivity detector. Dodecane concentration was assessed using a GC equipped with a flame ionized detector (FID) (Agilent Technologies, Santa Clara, USA) and a HP-5 capillary column (30 m; 0.25 mm), set for an isothermal run at 100 °C for 5 min. The injector (splitless mode) was set at 270 °C, while the FID was kept at 320 °C; the carrier gas (N_2_) flow rate was 60 mL min^−1^ and injected sample volume was 5 μL. Samples were prepared as follows: first, 0.7 mL of culture were removed from syringes and extracted from the water-phase using 1:1 n-hexane; then, they were vigorously shaken for 1 min and set aside for 15 min. The upper layer of hexane and extracted dodecane was collected and injected into the GC-FID. Ectoine was assessed according to Onraedt *et al*.^46^.

### Transcriptomic analysis

Ten independent cultures of *A. borkumensis* SK2 were grown at 0.1 and 10 MPa as described above. At the end of the experiments, HP was gently released and cultures pooled together for centrifugation within 5 min. Centrifuge was pre-refrigerated at 4 °C and cells centrifuged at 13000rpm for 5min (Sorval RC5c PLUS, Beckman, Suarlée, Belgium). Supernatant was discarded, RNAlater added (ThermoFischer, Gent, Belgium) and pellets stored at −80 °C for further RNA extraction.

### RNA extraction and QC

RNA was isolated from pelleted cells using the Rneasy Mini Kit (Qiagen, Antwerp, Belgium) following manufacturer’s instructions. On-column DNase digestion was performed during RNA extraction. RNA concentration was determined using the NanoDrop 2000 UV-Vis spectrophotometer (Thermo Scientific, Waltham, MA, USA). Pellets recovered after incubation at 0.1 and 10 MPa yielded 855.4 and 164.3 ng RNA/μL, respectively. RNA quality control was performed using the 2100 Bioanalyzer microfluidic gel electrophoresis system (Agilent Technologies, Santa Clara, USA).

### RNA library prep and sequencing

Libraries for RNA-sequencing were prepared using the ScriptSeq Complete (Bacteria) sample prep kit (Epicentre – Illumina, San Diego, CA, USA). Starting material (1 μg) of total RNA was depleted of rRNAs using Ribo-Zero magnetic bead based capture-probe system (Illumina, Hayward, USA). Remaining RNA (including mRNAs, lin-cRNAs and other RNA species) was subsequently purified (Agencourt RNA- Clean XP, Beckman Coulter, Brea, CA, USA) and fragmented using enzymatic fragmentation. First and second strand synthesis were performed and double stranded cDNA was purified (AgencourtAMPure XP). RNA stranded libraries were pre-amplified and purified (AgencourtAMPure XP). Library size distribution was validated and quality inspected using the 2100 Bioanalyzer (high sensitivity DNA chip, Agilent Technologies). High quality libraries were quantified using the Qubit Fluorometer (Life Technologies, Carlsbad, CA, USA), concentration normalized and samples pooled according to number of reads. Sequencing was performed on a NextSeq500 instrument using Mid Output sequencing kit (150 cycles) according to manufacturer’s instructions (Illumina).

### Data processing workflow

Data analysis pipeline was based on the Tuxedo software package. Components of the RNA-seq analysis pipeline included Bowtie2 (v. 2.2.2), TopHat (v2.0.11) and Cufflinks (v2.2.1) and are described in detail below. TopHat is a fast splice junction mapper for RNA-Seq reads, which aligns sequencing reads to the reference genome using the sequence aligner Bowtie2. It uses sequence alignments to identify splice junctions for both known and novel transcripts. Cufflinks takes the alignment results from TopHat to assemble the aligned sequences into transcripts, constructing a map of the transcriptome, based on a previously reported transcriptome annotation^69^.

### Data analysis

Genes were grouped according to orthologous clusters using the database provided by Ortholuge DB^70^. Only clusters classified as supporting-species-divergence (SSD) and Borderline-SSD were considered and the rest were discarded (Divergent-SSD, Similar Non-SSD and unevaluated orthologs [RBB]). Up and downregulation analysis was expressed on a log2 basis, indicating fold changes in fragments per kilobase of transcript per million mapped reads (FPKM) between samples at 0.1 and 10 MPa. Gene clusters were arbitrarily considered up-regulated under HP when their log2 fold change was higher than 0.5 between 0.1 and 10 MPa. On the contrary, it was considered that downregulated genes had a −0.50 fold change. All gene clusters that were expressed between −0.5 and 0.5 were considered to be unaffected by the increase in HP. Hence, the ±0.5 log2 fold change was established in order to have a reasonable compromise in the definition of both upregulated and unaffected genes, provided that a higher threshold may be more appropriate to assess upregulation but would result into an overestimation of unaffected genes. Final analysis of up and down-regulated genes, and clusters of orthologous gene (COG) category was done using the database provided by KEGG (www.genome.jp/kegg).

### Statistical analysis

Results were expressed as mean values of experiments made in 4 to 20 independent replicates. Bars in the graphs indicate a 95% confidence interval (95% CI) calculated using a Student *t*-test with a two-sided distribution. Statistical significance was assessed using a nonparametric test (Mann-Whitney test) which considered a two-sided distribution with 95% CI.

## Additional Information

**How to cite this article**: Scoma, A. *et al*. An impaired metabolic response to hydrostatic pressure explains *Alcanivorax borkumensis* recorded distribution in the deep marine water column. *Sci. Rep.* **6**, 31316; doi: 10.1038/srep31316 (2016).

## Supplementary Material

Supplementary Information

## Figures and Tables

**Table 1:** Expression of genes related with alkane and fatty acid metabolism in *A. borkumensis* SK2 under 0.1 and 10 MPa.

Pathway	Regulation	log2 FC	10 MPa	0.1 MPa	Cluster ID	Locus Tag	Description
*Fatty acid metabolism*							
	+	1.89	1557.7	421.7	764	ABO_0201	cytochrome P450 family protein
	+	1.78	1183.1	345.7	764	ABO_2288	cytochrome P450
	+	1.70	834.6	257.1	914	ABO_0203	FAD-dependent oxidoreductase family protein
	+	1.10	867.5	405.8	407	ABO_0122	alkane-1-monooxygenase
	=	−0.20	37.0	42.5	169	ABO_0162	rubredoxin reductase
	=	−0.44	159.4	215.9	1408	ABO_1231	alcohol dehydrogenase
	−	−0.53	43.3	62.5	1119	ABO_0117	alcohol dehydrogenase/ formaldehyde dehydrogenase
	−	−0.64	106.3	166.2	30	ABO_0061	alcohol dehydrogenase
	−	−0.99	31.4	62.5	696	ABO_2483	alcohol dehydrogenase
	−	−1.54	28.1	81.7	2020	ABO_0962	aldehyde dehydrogenase family protein
	−	−1.65	55.3	173.5	1325	ABO_2384	cytochrome P450
*Fatty acid biosynthesis*							
	+	0.97	190.9	97.2	1167	ABO_1071	fabF; 3-oxoacyl-[acyl-carrier-protein] synthase
	+	0.77	374.4	219.1	2166	ABO_1154	FabZ; (3R)-hydroxymyristoyl-[acyl carrier protein] dehydratase
	+	0.76	264.6	156.2	299	ABO_0835	fabA; 3-hydroxydecanoyl-(acyl carrier protein) dehydratase
	+	0.57	136.3	91.8	49	ABO_1069	fabG; 3-oxoacyl-(acyl-carrier-protein) reductase
	=	0.12	113.6	104.5	1382	ABO_0834	3-oxoacyl-[acyl-carrier-protein] synthase
	=	−0.17	43.0	48.5	2093	ABO_1215	enoyl-[acyl-carrier-protein] reductase
	−	−3.48	87.5	979.0	1649	ABO_1713	3-ketoacyl-(acyl-carrier-protein) reductase
*Fatty acid degradation*							
	=	0.50	100.8	71.5	143	ABO_1653	3-ketoacyl-CoA thiolase
	=	−0.07	104.4	109.9	1313	ABO_1652	multifunctional fatty acid oxidation complex subunit alpha
	=	−0.40	66.1	86.9	1467	ABO_0957	acyl-CoA dehydrogenase
	-	−0.67	59.6	94.9	660	ABO_1566	fatty oxidation complex subunit alpha
	−	−0.71	83.2	135.9	644	ABO_0571	acyl-CoA dehydrogenase
	−	−1.55	30.6	89.5	1976	ABO_0253	acetyl-CoA acyltransferase
	−	−1.55	19.4	56.9	689	ABO_1702	acyl-CoA dehydrogenase middle domain-containing protein
	−	−1.70	52.0	168.7	55	ABO_1772	acyl-CoA dehydrogenase
	−	−2.57	77.6	459.3	1865	ABO_1121	acyl-CoA dehydrogenase

**Table 2:** Gene expression of glyoxylate and TCA cycle, ATP synthase subunits, cytochromes C and B, and alternative respiration pathways in *A. borkumensis* SK2 cells grown under 10 MPa as compared to 0.1 MPa.

Pathway	Regulation	log2 FC	10 MPa	0.1 MPa	Cluster ID	Locus Tag	Description
*Glyoxylate cycle*							
	+	2.46	1080.6	196.5	1810	ABO_2741	isocitrate lyase
	+	1.98	224.4	56.9	1871	ABO_1201	bifunctional aconitate hydratase 2/2-methylisocitrate dehydratase
	+	1.69	312.3	97.0	2127	ABO_1267	malate synthase G
	=	0.22	132.7	113.8	338	ABO_1248	malate dehydrogenase
	=	0.08	169.1	160.3	1864	ABO_1501	type II citrate synthase
	=	−0.12	82.3	89.3	1152	ABO_1431	aconitate hydratase
	−	−1.27	321.6	778.2	872	ABO_0694	aconitate hydratase
*TCA cycle*							
	+	1.98	224.4	56.9	1871	ABO_1201	bifunctional aconitate hydratase 2/2-methylisocitrate dehydratase
	+	1.15	186.7	84.2	1441	ABO_0296	isocitrate dehydrogenase
	+	0.92	559.4	295.7	1344	ABO_1493	succinyl-CoA synthetase subunit beta
	+	0.79	77.5	45.0	691	ABO_1540	fumarate hydratase
	+	0.73	75.5	45.4	2058	ABO_0275	phosphoenolpyruvate carboxykinase
	+	0.53	591.8	409.7	2110	ABO_1496	2-oxoglutarate dehydrogenase E1 component
	=	0.26	93.9	78.4	247	ABO_1497	succinate dehydrogenase, iron-sulfur
	=	0.22	132.7	113.8	338	ABO_1248	malate dehydrogenase
	=	0.08	169.1	160.3	1864	ABO_1501	type II citrate synthase
	=	−0.01	282.6	284.6	991	ABO_1495	dihydrolipoamide succinyltransferase
	=	−0.04	98.0	100.8	475	ABO_1498	succinate dehydrogenase flavoprotein subunit
	=	−0.04	157.3	161.3	486	ABO_1499	succinate dehydrogenase, hydrophobic membrane anchor protein
	=	−0.05	307.8	318.3	1918	ABO_1492	succinyl-CoA synthetase subunit alpha
	=	−0.08	282.6	298.4	1943	ABO_1494	2-oxoglutarate dehydrogenase lipoamide dehydrogenase component
	=	−0.11	121.7	131.6	1539	ABO_2749	fumarate hydratase
	=	−0.12	82.3	89.3	1152	ABO_1431	aconitate hydratase
	=	−0.48	182.4	253.9	1044	ABO_0622	pyruvate dehydrogenase subunit E1
	=	−0.5	110.3	156.3	1267	ABO_1282	isocitrate dehydrogenase
	−	−0.56	346.8	512.4	1237	ABO_1500	succinate dehydrogenase, cytochrome b556 subunit
	−	−0.76	162.4	275.5	128	ABO_0623	pyruvate dehydrogenase, E2 component
	−	−1.27	321.6	778.2	872	ABO_0694	aconitate hydratase
*ATP synthase*							
	+	3.38	1345.4	129.3	506	ABO_2730	atpF; ATP synthase F0 subunit B
	+	3.08	332.4	39.4	942	ABO_2729	atpH; ATP synthase subunit delta
	+	1.70	521.2	160.6	163	ABO_2728	atpA; ATP synthase subunit alpha
	+	1.28	304.1	125.5	485	ABO_2727	atpG; ATP synthase subunit gamma
	+	1.07	599.1	286.3	323	ABO_2732	atpB; F0F1 ATP synthase subunit A
	+	0.95	321.1	166.7	1566	ABO_2726	atpD; F0F1 ATP synthase subunit beta
	+	0.77	325.4	191.4	1581	ABO_2725	atpC; ATP synthase subunit epsilon
	+	0.73	340.4	204.8	1637	ABO_2733	atpI; ATP synthase subunit I
	−	−0.69	35.1	56.5	1610	ABO_2731	ATP synthase F0 subunit C
*Energy Production (alternative respiration*)							
	+	1.10	196.8	91.9	2010	ABO_1032	Na(+)-translocating NADH-quinone reductase subunit A
	+	1.02	393.5	194.5	359	ABO_1034	Na(+)-translocating NADH-quinone reductase subunit C
	=	0.19	205.1	179.2	337	ABO_1037	Na(+)-translocating NADH-quinone reductase subunit F
	=	−0.08	149.1	157.8	1158	ABO_1033	Na(+)-translocating NADH-quinone reductase subunit B
	=	−0.23	92.8	108.7	615	ABO_1035	Na(+)-translocating NADH-quinone reductase subunit D
	−	−2.11	46.1	198.7	781	ABO_1036	Na(+)-translocating NADH-quinone reductase subunit E
*Cytochrome C reductase*							
	+	1.45	241.3	88.1	1648	ABO_0578	ubiquinol–cytochrome c reductase, iron-sulfur subunit
	+	1.17	437.0	194.1	1172	ABO_0580	ubiquinol-cytochrome c reductase cytochrome c1 subunit
	=	0.00	267.1	268.0	285	ABO_0579	ubiquinol-cytochrome c reductase cytochrome subunit B
*Cytochrome C oxidase*							
	+	0.89	201.5	108.5	1142	ABO_1900	cytochrome c oxidase subunit II, CoxB
	=	0.01	201.0	199.7	947	ABO_1897	cytochrome c oxidase subunit III, CoxC
	=	−0.07	120.4	126.5	1852	ABO_1899	cytochrome c oxidase subunit I, CoxA
	−	−0.58	99.0	148.0	1941	ABO_1905	cytochrome c oxidase assembly protein, CtaA
	−	−1.04	106.6	219.2	1533	ABO_1898	cytochrome c oxidase assembly protein, CtaG
	−	−1.61	57.2	174.9	447	ABO_2036	cytochrome c oxidase subunit I, CyoB
	−	−1.86	44.6	161.5	1213	ABO_2037	cytochrome o ubiquinol oxidase subunit II, CyoA
	−	−2.13	19.0	83.1	955	ABO_2035	cytochrome o ubiquinol oxidase subunit III, CyoC
	−	−2.41	90.6	480.1	398	ABO_2034	cytochrome o ubiquinol oxidase, protein CyoD
*Other Cytochromes C and B*							
	+	3.18	2551.5	280.9	1326	ABO_2651	cytochrome c-type protein
	+	2.13	173.7	39.7	288	ABO_2540	cytochrome c5
	+	1.65	374.4	119.4	1440	ABO_2650	cytochrome c4
	+	0.57	80.7	54.4	864	ABO_2539	cytochrome c5
	=	−0.39	72.1	94.3	611	ABO_0838	cytochrome c biogenesis protein, CcmH
	−	−1.02	58.4	118.4	1697	ABO_0839	cytochrome c-type biogenesis protein
	−	−1.04	54.6	112.1	386	ABO_0874	cytochrome c-type biogenesis protein, CcmE
	−	−1.75	24.7	83.0	2031	ABO_1185	cytochrome c family protein
	−	−1.83	30.0	106.6	421	ABO_0836	cytochrome c-type biogenesis protein CcmF
	−	−2.59	109.9	663.6	1794	ABO_0099	cytochrome B651

**Table 3:** Gene expression in amino acid and cell division pathways in *A. borkumensis* SK2 cells grown under 10 MPa as compared to 0.1 MPa.

Pathway	Regulation	log2 FC	10 MPa	0.1 MPa	Cluster ID	Locus Tag	Description
*Glycine, Serine and Threonine Metabolism*							
	+	1.97	337.3	86.4	1005	ABO_2594	glycine cleavage system T protein
	+	1.37	590.4	228.6	1355	ABO_0807	threonine synthase
	+	1.22	125.4	53.9	222	ABO_2176	serine hydroxymethyltransferase
	+	0.79	160.3	93.0	1666	ABO_2593	glycine cleavage system H protein
	+	0.76	136.6	80.6	709	ABO_2442	phosphoserine phosphatase
	+	0.71	135.0	82.4	1054	ABO_0806	homoserine dehydrogenase
	+	0.51	147.1	103.5	360	ABO_1769	phosphoglycerate mutase
	=	0.46	40.7	29.5	136	ABO_2592	glycine dehydrogenase subunit 1
	=	0.45	127.7	93.7	223	ABO_0688	TRAP dicarboxylate transporter
	=	0.3	24.3	19.8	1494	ABO_2591	glycine dehydrogenase subunit 2
	=	0.18	92.3	81.4	149	ABO_1436	phosphoserine phosphatase
	=	-0.02	169.2	171.2	1271	ABO_0042	phosphotransferase family protein
	=	−0.08	282.6	298.4	1943	ABO_1494	2-oxoglutarate dehydrogenase lipoamide
	=	−0.23	89.2	104.5	490	ABO_2605	threonine dehydratase, biosynthetic
	=	−0.23	89.2	104.5	490	ABO_2605	threonine dehydratase, biosynthetic
	−	−0.61	76.1	116.1	1056	ABO_1750	phosphoserine aminotransferase
*L-leucine Biosynthesis*							
	+	4.91	3676.8	122.4	510	ABO_2437	ilvE; branched-chain amino acid aminotransferase
	+	2.45	159.5	29.2	1318	ABO_2301	ilvE; branched-chain amino acid aminotransferase
	+	1.53	833.4	289.3	2145	ABO_1470	leuC; isopropylmalate isomerase large subunit
	+	1.00	674.3	337.8	559	ABO_0482	ilvH; acetolactate synthase 3 regulatory subunit
	+	0.81	122.0	69.5	1715	ABO_0481	ilvB-1; acetolactate synthase 3 catalytic subunit
	=	0.39	215.5	164.5	595	ABO_0485	ketol-acid reductoisomerase
	=	0.27	43.4	36.0	394	ABO_2312	dihydroxy-acid dehydratase
	=	0.23	272.9	232.5	339	ABO_1467	3-isopropylmalate dehydrogenase
	=	0.20	436.2	378.6	1492	ABO_1469	3-isopropylmalate dehydratase small subunit
	=	0.11	86.9	80.3	1428	ABO_0638	2-isopropylmalate synthase
*Valine, Leucine and Isoleucine Biosynthesis*							
	+	4.91	3676.8	122.4	510	ABO_2437	2-isopropylmalate synthase
	+	2.45	159.5	29.2	1318	ABO_2301	branched-chain amino acid aminotransferase
	+	1.53	833.4	289.3	2145	ABO_1470	isopropylmalate isomerase large subunit
	+	1	674.3	337.8	559	ABO_0482	acetolactate synthase 3 regulatory subunit
	+	0.81	122.0	69.6	1715	ABO_0481	acetolactate synthase 3 catalytic subunit
	=	0.39	215.5	164.5	595	ABO_0485	ketol-acid reductoisomerase
	=	0.27	43.4	36.0	394	ABO_2312	dihydroxy-acid dehydratase
	=	0.23	272.9	232.5	339	ABO_1467	3-isopropylmalate dehydrogenase
	=	0.2	436.2	378.6	1492	ABO_1469	3-isopropylmalate dehydratase small subunit
	=	0.11	86.9	80.3	1428	ABO_0638	2-isopropylmalate synthase
	=	−0.23	89.2	104.5	490	ABO_2605	threonine dehydratase, biosynthetic
	−	−0.53	62.3	89.9	1571	ABO_0180	dihydroxy-acid dehydratase
	−	−0.78	46.8	80.4	2136	ABO_0704	acetolactate synthase
*Cell division*							
	+	1.44	461.9	170.6	1439	ABO_0591	cell division protein FtsL
	+	0.78	287.3	166.8	1367	ABO_0948	cell division protein ZipA
	+	0.75	69.9	41.5	2142	ABO_2566	cell division protein FtsY
	=	0.36	165.4	128.5	778	ABO_1290	cell division protein FtsK
	=	−0.07	843.5	882.6	1176	ABO_0322	cell division protein FtsH
	=	−0.07	65.9	69.4	2138	ABO_0603	cell division protein FtsZ
	−	−0.86	15.1	27.5	1319	ABO_2568	cell division protein FtsX
	−	−0.91	52.6	98.8	1374	ABO_0601	cell division protein FtsQ
	−	−0.94	88.6	169.5	539	ABO_0597	cell division protein FtsW
	−	−1.05	13.4	27.8	1874	ABO_2567	cell division ATP-binding protein FtsE
	−	−1.66	34.0	107.6	449	ABO_0602	cell division protein FtsA

**Table 4:** Gene expression in the ectoine biosynthesis pathway in *A. borkumensis* SK2 cells grown under 10 MPa as compared to 0.1 MPa.

Pathway	Regulation	log2 FC	10 MPa	0.1 MPa	Cluster ID	Locus Tag	Description
*Ectoine biosynthesis*							
	+	2.90	704.4	94.5	661	ABO_2150	ectA; DABA acetyltransferase
	+	2.26	1697.1	355.1	471	ABO_2152	ectC; L-ectoine synthase
	+	2.07	538.8	128.4	1377	ABO_1797	lysC; aspartokinase
	+	1.90	321.4	86.1	1212	ABO_2151	ectB; diaminobutyrate–2-oxoglutarate aminotransferase
	+	0.76	553.4	325.7	628	ABO_1466	asd-2; aspartate-semialdehyde dehydrogenase
