# Supplementary material for: An impaired metabolic response to hydrostatic pressure explains Alcanivorax borkumensis recorded distribution in the deep marine water column
Source: Sci Rep. 2016 Aug 12;6:31316. doi: 10.1038/srep31316 (PMC4981847; doi:10.1038/srep31316)
Supplement: Supplementary Information [file srep31316-s1.pdf]

An impaired metabolic response to hydrostatic pressure explains *Alcanivorax borkumensis* recorded distribution in the deep marine water column

Alberto Scoma[1], Marta Barbato[2], Sara Borin[2], Daniele Daffonchio[2,3] and Nico Boon[1*]

[1] Center of Microbial Ecology and Technology (CMET), University of Gent, Coupure Links 653, B 9000 Gent, Belgium

[2] Department of Food, Environmental and Nutritional Sciences (DeFENS), University of Milano, Via Celoria 2, 20133, Milano, Italy

[3] Biological and Environmental Sciences and Engineering Division, King Abdullah University of Science and Technology (KAUST), 4700, Thuwal 23955-6900, Kingdom of Saudi Arabia

Corresponding Author:

*** Prof. Dr. Ir. Nico Boon**

CMET – Ugent, Coupure Links 653, 9000, Gent; Nico.Boon@UGent.be

Phone +32 (0)9 264 59 76; Fax +32 (0)9 264 62 48

Figure Legend

Figure S1. C12 bioavailability in *A. borkumensis* SK2 under atmospheric (0.1 MPa) and mild HP (5 and 10MPa). Initial C12 concentration provided was equal to 7.5 g L^{-1}. Bars indicate 95% confidence intervals.

Figure S2: Upregulated COG in *A. borkumensis* SK2 cells incubated at 10MPa with respect to expression levels at 0.1MPa. Percentage of expression is normalized on the total number of upregulated gene clusters (354/2202, 16%).

Table Legend

Table S1. Expression of genes related with the gluconeogenesis and pentose phosphate pathway connecting the TCA cycle to the purine, pirimidine and histidine metabolism in *A. borkumensis* SK2 cells grown under 10MPa as compared to 0.1MPa

Table S2. Expression of genes related with transcription in *A. borkumensis* SK2 cells grown under 10MPa as compared to 0.1MPa

Table S3. Expression of genes related with DNA synthesis and repair in *A. borkumensis* SK2 cells grown under 10MPa as compared to 0.1MPa

Table S4. Expression of genes related with protein translation in *A. borkumensis* SK2 cells grown under 10MPa as compared to 0.1MPa

Table S5. Expression of genes related with 30S and 50S ribosomal subunits in *A. borkumensis* SK2 cells grown under 10MPa as compared to 0.1MPa

Table S6. Expression of genes related with some typical pressure-responsive genes in *A. borkumensis* SK2 cells grown under 10MPa as compared to 0.1MPa

Table S7. Expression of genes related with biotin biosynthetic pathway in *A. borkumensis* SK2 cells grown under 10MPa as compared to 0.1MPa

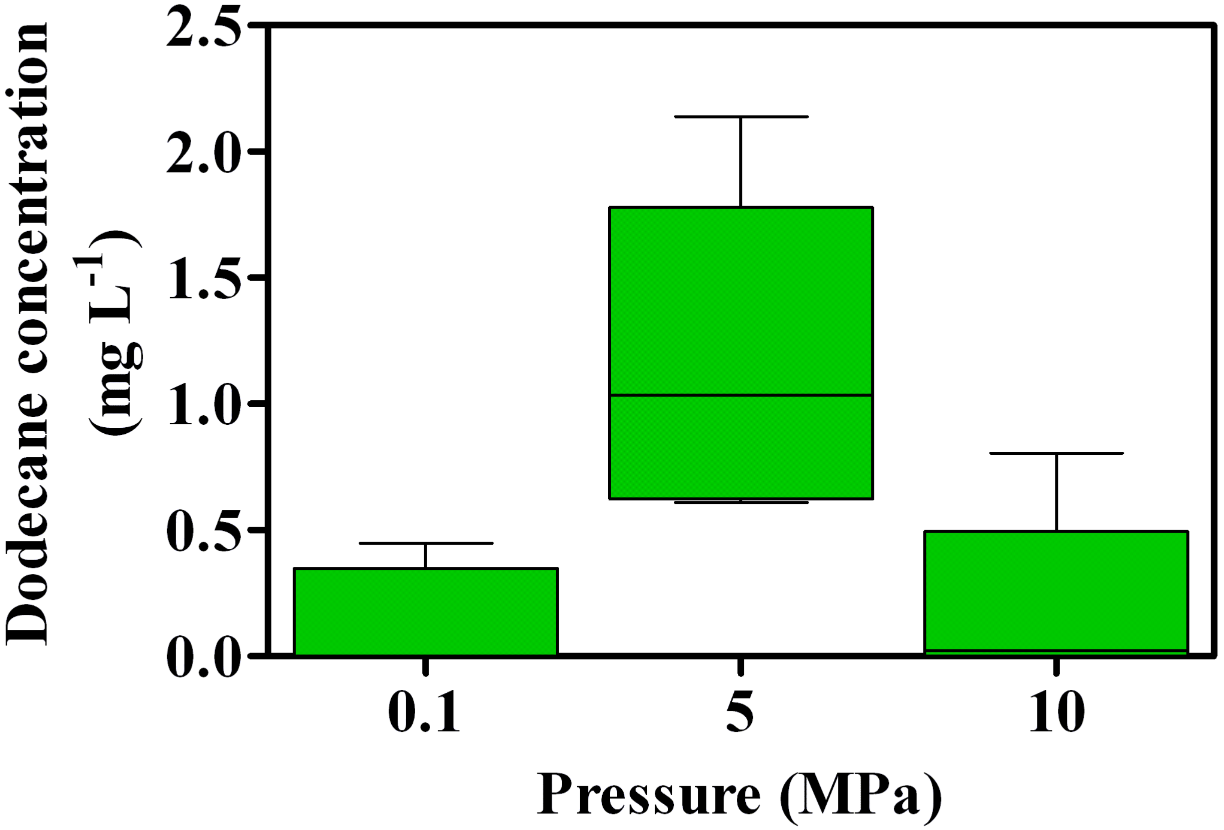

Dodecane concentration
(mg L-1)
2.5
2.0
1.5
1.0
0.5
0.0
0.1
5
10
Pressure (MPa)

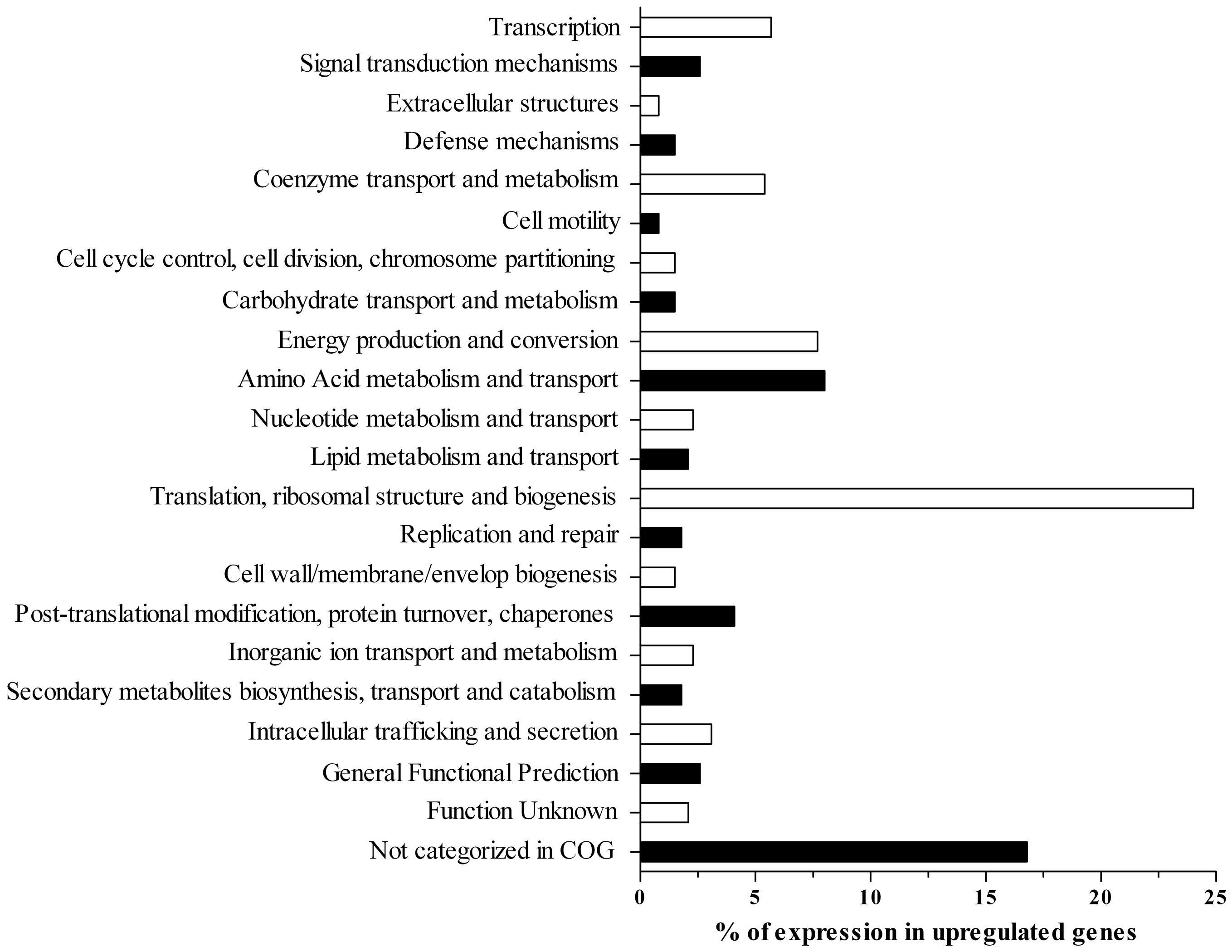
Transcription
Signal transduction mechanisms
Extracellular structures
Defense mechanisms
Coenzyme transport and metabolism
Cell motility
Cell cycle control, cell division, chromosome partitioning
Carbohydrate transport and metabolism
Energy production and conversion
Amino Acid metabolism and transport
Nucleotide metabolism and transport
Lipid metabolism and transport
Translation, ribosomal structure and biogenesis
Replication and repair
Cell wall/membrane/envelop biogenesis
Post-translational modification, protein turnover, chaperones
Inorganic ion transport and metabolism
Secondary metabolites biosynthesis, transport and catabolism
Intracellular trafficking and secretion
General Functional Prediction
Function Unknown
Not categorized in COG
0
5
10
15
20
25
% of expression in upregulated genes

Table S1. Expression of genes related with the gluconeogenesis and pentose phosphate pathway connecting the TCA cycle to the purine, pirimidine and histidine metabolism in *A. borkumensis* SK2 cells grown under 10MPa as compared to 0.1MPa

**Pathway**	**Regulation**	**log2 FC**	**10MPa**	**0.1MPa**	**Cluster ID**	**Locus Tag**	**Description**
Glycolysis/Gluconeogenesis							
(connection between TCA and Pentose pathway)	+	1.31	614.3	248.1	922	ABO_1031	glyceraldehyde-3-phosphate dehydrogenase
	+	0.73	75.45	45.37	2058	ABO_0275	phosphoenolpyruvate carboxykinase
	+	0.51	147.09	103.46	360	ABO_1769	phosphoglycerate mutase
	=	0.26	169.44	141.78	321	ABO_2613	phosphoglycerate kinase
	=	0.12	203.26	186.56	1268	ABO_1164	enolase
	-	-0.91	30.04	56.43	905	ABO_2614	glyceraldehyde 3-phosphate dehydrogenase
Pentose Phosphate Pathway							
(connection between Glycolysis and Purine/Pyridine/Histidine metabolism)	+	0.85	80.36	44.62	75	ABO_2606	ribose 5-phosphate isomerase
	=	-0.1	55.37	59.48	1845	ABO_2615	transketolase
	=	-0.12	59.79	65.06	2084	ABO_0518	ribose-phosphate pyrophosphokinase
	=	-0.41	112.52	149.47	1262	ABO_0937	phosphomannomutase
	=	-0.48	31.24	43.48	1950	ABO_2042	ribulose-phosphate 3-epimerase

Table S2. Expression of genes related with transcription in *A. borkumensis* SK2 cells grown under 10MPa as compared to 0.1MPa

Pathway	Regulation	log2 FC	10MPa	0.1MPa	Cluster ID	Locus Tag	Description
Transcriptional regulator							
	+	1.42	91.7	34.2	2143	ABO_0036	transcriptional regulator PyrR
	+	1.39	314.0	120.1	1630	ABO_1434	GntR family transcriptional regulator
	+	1.35	557.7	218.9	1700	ABO_2750	TetR family transcriptional regulator
	+	1.00	153.8	76.8	1695	ABO_1718	TetR family transcriptional regulator
	+	0.81	510.0	291.2	1815	ABO_0121	GntR family transcriptional regulator
	+	0.81	460.3	263.2	335	ABO_1082	TetR family transcriptional regulator
	+	0.70	253.3	156.1	117	ABO_2418	TetR family transcriptional regulator
	+	0.58	311.7	209.1	1957	ABO_0490	TetR family transcriptional regulator
	+	0.55	337.7	230.8	714	ABO_1835	transcriptional regulator
	+	0.51	498.3	350.5	1991	ABO_2014	Fis, Fis family transcriptional regulator
	=	0.45	450.5	330.8	1975	ABO_2175	transcriptional regulator
	=	0.28	239.7	197.1	825	ABO_0088	TetR family transcriptional regulator
	=	0.19	102.5	89.9	215	ABO_2116	LysR family transcriptional regulator
	=	0.16	200.1	179.1	1768	ABO_2149	MarR family transcriptional regulator
	=	0.14	307.9	278.6	2038	ABO_2120	transcriptional regulator
	=	-0.13	78.7	86.1	1144	ABO_0484	LysR family transcriptional regulator IlvY
	=	-0.21	835.9	969.5	1586	ABO_0309	transcriptional regulator Fur
	-	-0.51	112.4	160.0	1316	ABO_1437	transcriptional regulator CysB
	-	-0.57	292.6	435.1	2060	ABO_0782	GntR family transcriptional regulator
	-	-0.65	245.4	386.0	627	ABO_0054	TetR family transcriptional regulator
	-	-0.70	242.5	393.4	181	ABO_0198	TetR family transcriptional regulator
	-	-0.72	70.5	116.2	526	ABO_1241	transcriptional regulator
	-	-0.74	54.9	91.7	1731	ABO_2170	transcriptional terminator nusB
	-	-0.82	87.5	154.0	1922	ABO_2154	transcriptional regulator

							DegU
	-	-0.94	30.7	58.7	200	ABO_0019	transcriptional regulator MmsR
	-	-0.99	48.2	95.6	1615	ABO_1353	LysR family transcriptional regulator
	-	-1.00	41.8	83.6	1552	ABO_1600	transcriptional regulator Anr
	-	-1.05	73.0	151.6	2019	ABO_1016	TetR family transcriptional regulator
	-	-1.07	13.0	27.3	1096	ABO_0115	transcriptional regulator
	-	-1.10	35.4	75.9	1118	ABO_1842	MarR family transcriptional regulator
	-	-1.11	31.0	67.1	1066	ABO_0028	LysR family transcriptional regulator
	-	-1.15	54.0	120.0	1314	ABO_1341	transcriptional regulator Anr
	-	-1.30	75.9	187.3	196	ABO_1647	TetR family transcriptional regulator
	-	-1.34	29.2	74.1	147	ABO_0285	transcriptional regulator
	-	-1.42	61.4	164.4	1016	ABO_2438	transcriptional regulator
	-	-1.44	23.3	63.4	69	ABO_0118	LysR family transcriptional regulator
	-	-1.47	21.9	60.7	706	ABO_1405	MerR family transcriptional regulator
	-	-1.65	47.4	148.9	1334	ABO_2123	LysR family transcriptional regulator
	-	-1.77	105.0	359.0	422	ABO_0966	transcriptional regulator TtgR
	-	-1.83	51.0	181.2	1292	ABO_0992	TetR family transcriptional regulator
	-	-1.90	46.9	175.8	1472	ABO_0146	LysR family transcriptional regulator
	-	-2.03	18.1	74.1	1455	ABO_1706	TetR family transcriptional regulator
	-	-2.18	11.9	54.2	1084	ABO_2402	LysR family transcriptional regulator
	-	-2.52	29.4	168.5	275	ABO_1471	LysR family transcriptional regulator
	-	-2.92	16.4	124.5	976	ABO_0244	MerR family transcriptional regulator
	-	-3.31	65.8	653.8	1928	ABO_0031	transcriptional regulator QseB
Transcriptional elongators, activators, termination							
	+	1.60	149.6	49.3	2111	ABO_2463	transcription termination factor Rho
	+	1.57	914.0	308.9	751	ABO_0373	transcription antitermination protein NusG
	+	1.46	644.1	233.6	2066	ABO_0328	transcription elongation factor

	+	1.30	289.2	117.6	680	ABO_0319	transcription elongation factor GreA
	=	-0.23	145.3	170.6	2083	ABO_0369	transcriptional activator
	-	-0.69	95.0	152.8	585	ABO_2457	transcription elongation factor GreB
	-	-0.98	54.6	107.6	2103	ABO_0862	transcriptional activator MetR
	-	-1.22	21.9	51.1	722	ABO_1028	transcription-repair coupling factor

Table S3. Expression of genes related with DNA synthesis and repair in *A. borkumensis* SK2 cells grown under 10MPa as compared to 0.1MPa

Pathway	Regulation	log2 FC	10MPa	0.1MPa	Cluster ID	Locus Tag	Description
DNA polymerase							
	+	0.60	108.5	71.7	1331	ABO_1225	DNA polymerase III subunit epsilon
	=	0.50	71.7	50.7	9	ABO_1075	DNA polymerase III subunit delta'
	=	0.35	85.8	67.4	1713	ABO_1775	DNA polymerase III subunits gamma and tau
	=	0.09	311.3	292.7	1934	ABO_0002	DNA polymerase III subunit beta
	=	-0.38	67.6	88.0	542	ABO_1158	DNA polymerase III subunit alpha
	=	-0.50	126.0	178.1	2178	ABO_0659	DNA polymerase IV
	-	-0.55	36.0	52.6	1102	ABO_0494	DNA polymerase III subunit chi
	-	-0.80	26.3	45.9	1933	ABO_1949	DNA polymerase III subunit delta
	-	-1.23	27.0	63.4	1625	ABO_2659	DNA polymerase I
	-	-1.58	41.7	124.5	1088	ABO_2156	DNA polymerase III subunit epsilon
DNA repairing system							
	+	2.97	993.8	126.8	1956	ABO_1801	RecA, DNA repairing protein
	+	1.16	370.4	165.8	366	ABO_0003	RecF, DNA replication and repair protein RecF
	=	0.17	93.7	83.1	324	ABO_0310	RecN, DNA repair protein RecN
	=	-0.10	39.7	42.6	1708	ABO_1773	RecR, recombination protein RecR
	=	-0.18	52.8	59.7	1594	ABO_1292	RarA, recombination factor protein RarA
	-	-1.17	33.8	76.3	64	ABO_2413	RdgC, recombination associated protein
	-	-1.30	37.7	93.0	89	ABO_0214	RadC, DNA repair protein RadC-like protein
	-	-1.50	45.0	127.2	1965	ABO_2185	RadA, DNA repair protein RadA
	-	-1.83	23.9	84.7	1179	ABO_1629	RecO, DNA repair protein RecO

Table S4. Expression of genes related with protein translation in *A. borkumensis* SK2 cells grown under 10MPa as compared to 0.1MPa

**Pathway**	**Regulation**	**log2 FC**	**10MPa**	**0.1MPa**	**Cluster ID**	**Locus Tag**	**Description**
Translation factor							
	+	2.90	1490.8	199.1	930	ABO_1144	translation elongation factor Ts
	+	2.26	667.9	139.5	7	ABO_1841	translation initiation factor IF-3
	+	1.45	497.8	181.8	28	ABO_0329	translation initiation factor IF-2
	+	0.98	160.5	81.3	815	ABO_1286	translation initiation factor IF-1
	=	0.31	114.3	92.0	573	ABO_2446	translation elongation factor P
	-	-1.32	45.0	112.3	985	ABO_0684	translation elongation factor P
Elongation factors							
	+	2.38	1279.4	245.7	155	ABO_0383	Tuf, elongation factor Tu
	+	2.30	1213.9	245.9	155	ABO_0371	Tuf-1, elongation factor Tu
	+	0.55	238.7	163.5	1100	ABO_0382	FusA, elongation factor EF
RNA polymerase							
	+	1.49	425.9	151.2	739	ABO_0422	DNA-directed RNA polymerase subunit alpha
	+	0.55	208.8	142.4	1782	ABO_0378	DNA-directed RNA polymerase subunit beta
	+	0.58	224.4	150.0	1558	ABO_0379	DNA-directed RNA polymerase subunit beta'
	+	3.73	463.4	35.0	497	ABO_0177	DNA-directed RNA polymerase subunit omega
RNA methyltransferase							
	+	1.09	97.7	45.8	416	ABO_0813	RNA methyltransferase
	=	-0.45	55.7	75.9	1961	ABO_2192	RNA methyltransferase
	-	-1.08	78.5	166.0	302	ABO_0513	RNA methyltransferase
	-	-1.23	29.6	69.5	82	ABO_1624	RNA methyltransferase
	-	-2.04	8.1	33.6	400	ABO_2271	RNA methyltransferase
Pseudouridylate synthase							
	+	1.26	263.4	110.0	789	ABO_0331	pseudouridylate synthase
	=	0.16	28.5	25.5	489	ABO_1280	pseudouridylate synthase
	=	0.07	67.4	64.0	2	ABO_2537	pseudouridylate synthase

Table S5. Expression of genes related with 30S and 50S ribosomal subunits in *A. borkumensis* SK2 cells grown under 10MPa as compared to 0.1MPa

Pathway	Regulation	log2 FC	10MPa	0.1MPa	Cluster ID	Locus Tag	Description
Ribosomal proteins							
	+	4.99	5493.1	173.3	327	ABO_0453	50S ribosomal protein L27
	+	4.60	1969.1	81.2	466	ABO_0396	30S ribosomal protein S10
	+	4.39	1715.1	81.8	848	ABO_0576	50S ribosomal protein L13
	+	3.36	2078.6	202.2	1397	ABO_0397	50S ribosomal protein L3
	+	3.31	610.8	61.6	1111	ABO_0401	30S ribosomal protein S19
	+	3.23	747.4	79.8	1101	ABO_0215	50S ribosomal protein L28
	+	3.16	1081.2	121.0	1912	ABO_0402	50S ribosomal protein L22
	+	3.11	336.9	39.0	1183	ABO_0452	50S ribosomal protein L21
	+	2.98	785.7	99.8	917	ABO_2191	30S ribosomal protein S6
	+	2.96	1124.4	144.7	906	ABO_0408	50S ribosomal protein L24
	+	2.95	3254.4	420.1	1059	ABO_0416	50S ribosomal protein L15
	+	2.95	863.5	112.0	662	ABO_0404	50S ribosomal protein L16
	+	2.93	2418.1	317.5	1335	ABO_0411	30S ribosomal protein S8
	+	2.90	1449.5	193.6	66	ABO_0406	30S ribosomal protein S17
	+	2.89	4250.4	574.5	1903	ABO_0377	50S ribosomal protein L7/L12
	+	2.88	594.4	80.8	2053	ABO_1840	50S ribosomal protein L35
	+	2.80	2701.1	387.7	1541	ABO_0403	30S ribosomal protein S3
	+	2.78	1129.7	164.6	2176	ABO_0405	50S ribosomal protein L29
	+	2.78	489.0	71.3	180	ABO_0409	50S ribosomal protein L5
	+	2.77	255.9	37.5	1244	ABO_0517	50S ribosomal protein L25/general stress protein Ctc
	+	2.74	583.3	87.3	263	ABO_1143	30S ribosomal protein S2
	+	2.71	1347.3	206.3	151	ABO_0399	50S ribosomal protein L23
	+	2.61	570.2	93.6	2150	ABO_0414	30S ribosomal protein S5
	+	2.57	1063.4	179.3	251	ABO_0398	50S ribosomal protein L4
	+	2.40	2067.0	390.3	594	ABO_2240	50S ribosomal protein L31
	+	2.37	1722.3	332.6	334	ABO_0415	50S ribosomal protein L30
	+	2.25	237.5	49.9	1973	ABO_0407	50S ribosomal protein L14
	+	2.22	542.0	116.2	1254	ABO_0420	30S ribosomal protein S11
	+	2.20	764.9	166.8	1281	ABO_0412	50S ribosomal protein L6
	+	2.18	365.9	81.0	1365	ABO_2060	30S ribosomal protein S21
	+	2.18	786.9	174.3	1161	ABO_0400	50S ribosomal protein L2

+	2.14	846.0	191.9	1786	ABO_0332	30S ribosomal protein S15
+	2.11	698.6	162.2	1378	ABO_0376	50S ribosomal protein L10
+	2.07	151.7	36.1	1132	ABO_0456	30S ribosomal protein S20
+	1.81	548.2	156.1	1800	ABO_0374	50S ribosomal protein L11
+	1.78	1051.6	306.2	575	ABO_1745	30S ribosomal protein S1
+	1.70	891.2	275.0	94	ABO_0381	30S ribosomal protein S7
+	1.60	229.3	75.5	2106	ABO_0413	50S ribosomal protein L18
+	1.57	635.8	214.0	808	ABO_0801	50S ribosomal protein L19
+	1.34	395.0	155.9	1647	ABO_2189	30S ribosomal protein S18
+	1.06	145.5	69.8	1895	ABO_0419	30S ribosomal protein S13
+	0.99	85.6	43.2	1385	ABO_2188	50S ribosomal protein L9
+	0.98	233.7	118.3	2029	ABO_0380	30S ribosomal protein S12
+	0.94	202.3	105.5	1759	ABO_0421	30S ribosomal protein S4
+	0.88	103.9	56.6	258	ABO_0798	30S ribosomal protein S16
=	0.49	139.2	98.9	610	ABO_0375	50S ribosomal protein L1
=	0.42	66.5	49.7	913	ABO_0410	30S ribosomal protein S14
=	0.26	639.5	535.6	665	ABO_0216	50S ribosomal protein L33
=	0.21	303.1	261.4	1232	ABO_1066	50S ribosomal protein L32
=	0.00	52.2	52.1	2091	ABO_1839	50S ribosomal protein L20
=	-0.18	153.6	174.5	1225	ABO_0423	50S ribosomal protein L17
=	-0.42	161.0	215.6	1356	ABO_0577	30S ribosomal protein S9
-	-1.15	297.7	661.1	1929	ABO_0418	50S ribosomal protein L36

Table S6. Expression of genes related with some typical pressure-responsive genes in *A. borkumensis* SK2 cells grown under 10MPa as compared to 0.1MPa

**Pathway**	**Regulation**	**log2 FC**	**10MPa**	**0.1MPa**	**Cluster ID**	**Locus Tag**	**Description**
Sigma factors							
	+	1.57	3919.7	1319.4	474	ABO_0551	sigma 54 modulation protein/ribosomal protein S30EA
	-	-0.53	560.8	809.0	1579	ABO_2063	RNA polymerase sigma factor RpoD
	-	-0.57	80.2	118.9	1798	ABO_0552	RNA polymerase sigma-54 factor
	-	-0.79	3738.0	6459.0	1243	ABO_2569	RNA polymerase sigma-32 factor
	-	-1.36	62.1	159.1	73	ABO_0471	sigma-54 dependent DNA-binding response regulator
	-	-1.65	827.6	2606.1	798	ABO_1639	RNA polymerase sigma factor RpoE
	-	-1.88	9.8	36.1	363	ABO_2179	rna polymerase sigma-e factor
	-	-3.20	180.2	1653.3	464	ABO_1638	sigma factor algU negative regulatory protein AlgS
Chaperonines, CSP, HSP							
	+	1.15	725.3	327.4	633	ABO_2744	cold-shock domain-contain protein
	+	0.93	472.0	247.5	943	ABO_1283	cold-shock domain-contain protein
	=	0.39	68.0	51.9	2055	ABO_0277	heat shock protein 15 kDa
	=	-0.34	79.3	100.7	2011	ABO_0312	heat-inducible transcription repressor hrcA
	=	-0.38	445.9	581.5	920	ABO_0634	chaperonin, 60 kDa
	-	-0.91	232.3	437.9	1599	ABO_0313	heat shock protein GrpE
	-	-1.01	88.1	176.9	315	ABO_0276	chaperonin, 33 kDa
	-	-1.11	185.5	399.2	342	ABO_0315	heat shock protein DnaJ
	-	-1.47	150.5	416.5	572	ABO_1489	heat shock protein 90
	-	-1.54	8.8	25.5	1856	ABO_1912	cold-shock domain-contain protein
	-	-2.00	1796.3	7185.3	1897	ABO_0633	chaperonin, 10 kDa
	-	-2.16	175.9	784.8	2039	ABO_1180	heat shock protein HtpX
	-	-2.45	1092.7	5968.7	1277	ABO_1777	heat-shock protein IbpA
	-	-2.52	2017.7	11588.2	2001	ABO_0314	heat shock protein DnaK
Outer membrane proteins							
	+	2.76	6146.6	904.7	1985	ABO_1527	OmpA family protein
	=	0.22	471.6	404.7	531	ABO_1152	outer membrane protein OmpH
	=	0.16	174.3	155.6	1457	ABO_2500	outer membrane protein TolC
	=	-0.22	155.2	180.8	927	ABO_1151	outer membrane protein surface

						antigen family protein
=	-0.50	177.2	249.8	1785	ABO_1411	OmpA family protein
-	-0.80	32.4	56.5	1416	ABO_0520	outer membrane lipoprotein LolB
-	-0.87	177.2	324.2	1783	ABO_1291	outer membrane lipoprotein carri-er protein
-	-1.06	113.6	237.1	1981	ABO_0905	outer membrane polysaccharide export protein
-	-1.09	764.2	1628.9	1217	ABO_0822	outer membrane protein OprF
-	-1.11	140.1	302.8	1503	ABO_0308	outer membrane lipoprotein OmlA
-	-1.67	50.7	161.1	1227	ABO_1104	outer membrane siderophore re-ceptor
-	-1.73	32.1	106.7	1668	ABO_0076	outer membrane ferric siderophore receptor
-	-1.73	32.1	106.7	1668	ABO_0076	outer membrane ferric siderophore receptor
-	-1.86	33.0	119.5	860	ABO_2104	outer membrane phospholipase A
-	-2.00	41.8	167.6	768	ABO_1621	outer membrane porin
-	-2.34	147.6	745.5	613	ABO_1922	outer membrane protein W

Table S7. Expression of genes related with biotin biosynthetic pathway in *A. borkumensis* SK2 cells grown under 10MPa as compared to 0.1MPa

Pathway	Regulation	log2 FC	10MPa	0.1MPa	Cluster ID	Locus Tag	Description
Biotin synthesis							
	+	1.81	254.6	72.7529	1834	ABO_2220	biotin synthase
	+	0.97	190.86	97.21	1167	ABO_1071	3-oxoacyl-[acyl-carrier-protein] synthase
	+	0.77	374.35	219.1	2166	ABO_1154	(3R)-hydroxymyristoyl-[acyl carrier protein] dehydratase
	+	0.57	136.31	91.84	49	ABO_1069	3-oxoacyl-(acyl-carrier-protein) reductase
	=	0.29	46.3562	37.9769	434	ABO_2218	BioH, biotin biosynthesis protein bioH
	=	0.12	113.63	104.49	1382	ABO_0834	3-oxoacyl-[acyl-carrier-protein] synthase
	=	0.06	30.2729	28.9851	1348	ABO_2217	BioC, biotin biosynthesis protein BioC
	=	-0.17	43.03	48.51	2093	ABO_1215	enoyl-[acyl-carrier-protein] reductase
	-	-0.59	30.37	45.78	248	ABO_2219	8-amino-7-oxononanoate synthase
	-	-1.41	30.5	80.8	676	ABO_0256	7,8-diaminononanoate transaminase
	-	-1.61	15.63	47.55	670	ABO_2216	dethiobiotin synthase
	-	-3.48	87.54	978.96	1649	ABO_1713	3-ketoacyl-(acyl-carrier-protein) reductase
